# Inter-laboratory comparison of water solubility methods applied to difficult-to-test substances

**DOI:** 10.1186/s13065-021-00778-7

**Published:** 2021-09-15

**Authors:** Daniel J. Letinski, Aaron D. Redman, Heidi Birch, Philipp Mayer

**Affiliations:** 1 Toxicology and Environmental Sciences Division, ExxonMobil Biomedical Sciences, Inc., 1545 US Highway 22 East, Annandale, NJ 08801-3059 USA; 2 Department of Environmental Engineering, Technical University of Denmark, 2800 Kgs Lyngby, Denmark

**Keywords:** Water solubility, Ring test, Difficult-to-test, Slow-stir method, Generator column, Volatile, Hydrophobic organics

## Abstract

Water solubility is perhaps the single most important physical–chemical property determining the environmental fate and effects of organic compounds. Its determination is particularly challenging for compounds with extremely low solubility, frequently referred to as “difficult-to-test” substances and having solubility’s generally less than 0.1 mg/L. The existing regulatory water solubility test for these compounds is the column elution method. Its applicability, however, is limited, to non-volatile solid or crystalline hydrophobic organic compounds. There currently exists no test guideline for measuring the water solubility of very hydrophobic liquid, and potentially volatile, difficult-to-test compounds. This paper describes a “slow-stir” water solubility methodology along with results of a ring trial across five laboratories evaluating the method’s performance. The slow-stir method was applied to n-hexylcyclohexane, a volatile, liquid hydrophobic hydrocarbon. In order to benchmark the inter-laboratory variability associated with the proposed slow-stir method, the five laboratories separately determined the solubility of dodecahydrotriphenylene, a hydrophobic solid compound using the existing column elution guideline. Results across the participating laboratories indicated comparable reproducibility with relative standard deviations (RSD) of 20% or less reported for each test compound – solubility method pair. The inter-laboratory RSD was 16% for *n*-hexylcyclohexane (mean 14 µg/L, n = 5) using the slow-stir method. For dodecahydrotriphenylene, the inter-laboratory RSD was 20% (mean 2.6 µg/L, n = 4) using the existing column elution method. This study outlines approaches that should be followed and the experimental parameters that have been deemed important for an expanded ring trial of the slow-stir water solubility method.

**Supplementary Information:**

The online version contains supplementary material available at 10.1186/s13065-021-00778-7.

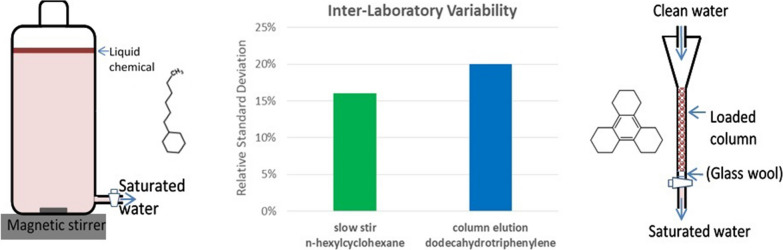

## Introduction

Aqueous solubility is a fundamental physical–chemical property that strongly influences the distribution, fate and effects of chemicals upon release into the environment [1]. Measured water solubility, along with other physical chemical properties (e.g. vapor pressure, octanol–water partition coefficient) are required as part of global chemical registrations. Low aqueous solubility can limit the aquatic toxicity potential of organic compounds [2, 3]. Regulators typically evaluate the aquatic toxicity of a substance in the context of its measured water solubility making accurate measurements critical for evaluating the reliability of these tests. Exposing test organisms to concentrations exceeding solubility can confound test interpretation in hazard and bioaccumulation assessments [4–6].

The most frequently cited test guideline for measuring water solubility to support chemical registrations and their associated hazard assessments is the OECD 105 Test Guideline [7]. However, this guideline only partially addresses the testing of so called “difficult-to-test” substances [8] for which the laboratory determination of water solubility can be particularly challenging. Difficult-to-test substances include pure compounds, isomeric mixtures and complex chemical mixtures of related compounds. They are typically very hydrophobic, have characteristically low water solubility (< 0.1 mg/L) and may also be volatile (high Henry’s Law constant) and biodegradable. A comprehensive review of issues associated with water solubility measurements covering a range of difficult-to-test substance categories is provided by Birch et al. [9]. That review also includes a decision tree to aid in selection of the most appropriate method for measuring a compound’s water solubility based on its physical state, relative hydrophobicity and other properties such as volatility.

The current OECD 105 guideline [7] is divided into two broad techniques based on whether the water solubility of an organic compound is anticipated to be greater or less than 10 mg/L. For more water soluble substances, the shake-flask method can be applied in which an excess of test chemical is equilibrated with water with rapid and robust agitation. The excess, undissolved chemical is physically separated from the saturated aqueous phase, typically by centrifugation or filtration, which in turn is analyzed for dissolved concentration using chemical specific methods. This technique suffers from two principal drawbacks making it unsuitable for very low-solubility substances. First, since agitation is vigorous, emulsion formation is problematic and confounds determination of the actual water solubility for hydrophobic liquids. Second, the agitation period is comparatively short and may be insufficient for substances to reach equilibrium solubility. The current OECD test guideline and corresponding U.S. EPA version [10] offer only a single method, the generator column (column elution) method for poorly soluble compounds. While this technique is intended for less water soluble compounds (i.e. < 10 mg/L), it is best suited for compounds that are solids at ambient temperature. Users are cautioned when using the generator column method for oily or liquid chemicals as the test compound may slough-off of the solid support phase resulting in emulsion formation in the water eluent. Another shortcoming is that volatile compounds may be lost during the time needed to collect the generator column fractions for analysis.

In order to fill the gap that currently exists for measuring the water solubility of very hydrophobic liquid compounds, this study describes a “slow-stir” method and the results of a ring trial test on a single difficult-to-test compound across five laboratories. Several researchers have previously applied this method to measure the water solubility of chemicals that are liquid, (semi-) volatile and hydrophobic [11–14]. This method is an adaption of the slow-stir technique initially developed to determine the octanol–water partition coefficients (K_ow_) of hydrophobic organic chemicals [15] and has been applied by a number of researchers to measure K_ow_. [16–19]. The slow-stir method for K_ow_ determination is recognized as an OECD test guideline [20].

The slow-stir water solubility method uses glass vessels, at least one liter in volume, to which reagent grade water is added. The typical bottle contains a sampling port or spigot at the bottom of the vessel and is filled to a water column height approximating that of the vessel’s tapered shoulders. Slow stir systems are depicted in Additional file 1: Figure S1. This configuration minimizes headspace but maximizes the test substance—water interface. The test compound is added in excess to the water surface at a loading generally three to four orders of magnitude greater than the expected water solubility. The top opening of the vessels is tightly sealed to minimize volatile loss of neat test substance from the system. The excess test substance provides a supply of free product to dissolve and, in the case of volatile substances, evaporate into the headspace. The system is mixed with a minimal vortex imparting just enough energy to move the small dollop of test substance and impart a slight dimple (< 0.5 cm) below the water surface. Care is taken to mix slowly so as to avoid emulsifying the test substance. Water samples are withdrawn from the bottom sampling port of the vessel, extracted and analyzed. As necessary, specific sampling techniques for dissolved volatile organics are used including: the use of gas tight syringes, minimizing sample transfer, and reduced storage duration prior to analysis. Sampling from slow-stir systems occurs over daily or weekly intervals until analysis indicates equilibrium has been reached representing maximum water solubility. Larger test (i.e. 8–20 L) vessels are generally used when the associated analytical method requires extraction of large water sample volumes and also provide a greater surface-to-volume ratio which can be advantageous, especially for surface active compounds. For substances where the solubility is extremely low (i.e. < 10 µg/L) lengthy equilibration periods (weeks-months) may be needed.

The slow-stir system described here depends on the test substance having a density of less than one gram per milliliter so as to be less than that of water, permitting the excess test substance to remain on the surface of the water column. For substances with a density greater than one, typically halogenated liquids, the excess test substance will reside on the bottom of the test vessel. For those substances, samples can be removed by pipette or siphon through the top of the vessel. Alternatively, a modified test vessel could be used where the sampling spigot is located higher up the side of the vessel to avoid sampling the denser-than-water test compound residing at the bottom of the vessel. The application of the slow-stir method to compounds with densities greater than one was not within the scope of the present study nor have been reported previously. This aspect of the slow-stir method will require additional study, perhaps as part of a broader ring test.

*n*-Hexylcyclohexane was selected as the model test compound for evaluation of the slow-stir method across five different laboratories. Hexylcyclohexane is a characteristic difficult-to-test liquid as it is very hydrophobic with very low aqueous solubility and is also fairly volatile. The ring trial also included the water solubility measurement of semi-volatile hydrophobic solid compound, dodecahydrotriphenylene using the existing column elution method. Since the two water solubility methods are applicable to hydrophobic organics differing in physical state, solids versus liquids, direct comparison of the two methods is not possible. Instead, a comparison is made of each water solubility method—compound pair using inter-laboratory variability as a proxy for method performance. An additional objective was to use the learnings from this ring-test to develop a detailed slow-stir method protocol for broader use and to facilitate a more comprehensive ring test with the ultimate goal of incorporation in the OECD 105 Test Guideline [7].

## Materials and methods

### Test compounds

The two difficult-to-test substances represent a very hydrophobic solid and liquid compound and are listed in Table 1. They were each obtained in high purity from commercial sources. Each participating laboratory obtained their own supply of test compound directly from the manufacturer.

**Table 1:** Test compounds, properties and sources

Compound (CAS)	Molecular Weight	Formula	Physical state^a^	Density^a^ (g/mL)	Log K_ow_^b,c^	Melting point (°C)	Boiling point (°C)	k_H_ atm-m^3^/mole^c,d^	Source	Purity %
Dodecahydrotriphenylene (1610-39-5)	240.4	C_18_H_24_	Solid	0.94	7.9	232	380	0.00151	Sigma-Aldrich	> 98.5
*n*-Hexylcyclohexane (4292-75-5)	168.3	C_12_H_24_	Liquid	0.8	6.5	− 43	221	0.289	TCI America	> 98.0

^a^At 20 °C

^b^Octanol–water partition coefficient

^c^EPISuite KOWWIN v1.67 estimates

d. Henry’s Law constant

### Participating laboratories

The five ring test participants are listed in Table 2. The laboratories represent two global petrochemical and chemical companies, two contract research organizations and a research institute. Three of the labs are located in Germany, one in the UK and one in the USA. Each lab has extensive experience conducting physical–chemical property testing including water solubility supported by analytical capabilities to perform trace level analysis.

**Table 2:** Laboratories participating in water solubility ring test

Laboratory	Location	Business
ExxonMobil Biomedical Sciences, Inc. (EMBSI)	USA	Global Petrochemical
BASF	Germany	Global Chemical
Fraunhofer Institute for Molecular Biology and Applied Ecology IME	Germany	Research Institution
Noack Laboratorien	Germany	Contract Research Organization
Smithers ERS Limited	UK	Contract Research Organization

### Water

Reagent grade water was used by each of the participating laboratories and included glass distilled water, double distilled water or Milli-Q^®^ demineralized water.

### Water solubility methods

Prior to initiation of the ring trial, each of the participating labs was provided the references for the applicable water solubility methods. These were the OECD 105 column elution method [7] and the two publications by Letinski et al. [11, 13] describing the slow-stir method. A preliminary meeting was held with representative from each of the labs to provide technical knowledge transfer of the slow-stir method and respond to any question the participants had regarding application of the method.

The column elution method as applied in this ring test is described in several regulatory test guidelines [7, 10]. The typical generator column consists of two concentric glass columns. The inner, narrow column is packed with test substance. The solid test substance is typically dissolved in a volatile solvent which is then pre-loaded onto an inert support phase (e.g. glass beads, diatomaceous earth, chromatographic support material) by rotary evaporation to coat the support phase and then remove the solvent. Alternatively, some practitioners load the column directly with ca. 0.5 g of neat test substance which is retained with plugs of fused silica wool. The outer column provides temperature control (20 °C) as it is connected to a recirculating water bath. Reagent grade water is pumped through the inner column contacting the test substance column at a low flow rate (i.e. < 1 mL/min). The water eluate is collected, extracted and analyzed. Consecutive fractions are collected until the concentration of test substance in water plateaus indicating equilibrium water solubility has been reached. The experiment is then repeated at a flow rate approximately one-half that applied in the first trial to confirm that equilibrium has been reached. In this ring test, two of the five participating labs applied dodecahydrotriphenylene dissolved in solvent to an inert support phase. The solvent was evaporated and the support phase coated with the test compounds. The remaining three laboratories packed the columns with neat test substance. Across the five laboratories, the column flow for the first run ranged from 0.4 to 0.8 mL/min. The volume of each fraction collected for analysis ranged from 5 to 25 mL. Details of the experimental parameters used by each of the participating labs in applying the generator column method are listed in Additional file 1: Table S1.

The slow-stir water solubility method is not currently cited in existing regulatory guidelines though a number of publications describe its application to difficult-to-test liquid compounds [11–14]. In this ring test, each laboratory employed all glass aspirator bottles with sampling spigots located toward the bottom of the vessels. Water volumes ranged from 0.8 to 4 L with initial vessel headspace accounting for 5 to 20% of the entire vessel volume. *n*-Hexylcyclohexane loadings ranged from 0.4 to 400 mg/L. The vessels were sealed with Teflon™, polypropylene, glass or high density polyethylene screw plugs or stoppers. Water was stirred at a rate imparting little to no visible vortex and stirring rates were estimated between 80 and 250 rpm. The specific experimental parameters used by each of the participating labs in applying the slow-stir method are listed in Additional file 1: Table S2.

### Analytical methods

The participating labs used a range of trace analytical methods to analyze concentrations of the two test substances in water across the two water solubility methods. The methods are summarized in Table 3.

**Table 3:** Summary of analytical methods used for water solubility determinations

Lab	Dodecahydrotriphenylene (column elution)	Hexylcyclohexane (slow-stir)
EMBSI	direct immersion SPME GC–MS	Static headspace-Trap GC-FID
BASF	Solvent extraction (dichloromethane), exchanged to methanol. HPLC–UV	Headspace SPME GC–MS
Fraunhofer Institute	Solvent extraction (cyclohexane), GC–MS	Solvent extraction (cyclohexane) GC–MS
Noack Laboratorien	Solvent extraction (cyclohexane) GC–MS	Solvent extraction (cyclohexane) GC–MS,
Smithers ERS Limited	Solvent extraction (hexane), tenfold concentration. GC–MS	Headspace SPME GC–MS

*SPME *solid phase microextraction using a 30 µm PDMS fiber (Supleco), *GC–MS* gas chromatography-mass spectrometry, *GC-FID *gas chromatography-flame ionization detection, *HPLC–UV* high performance liquid chromatography–ultraviolet detection

## Results

**Fig. 1:**
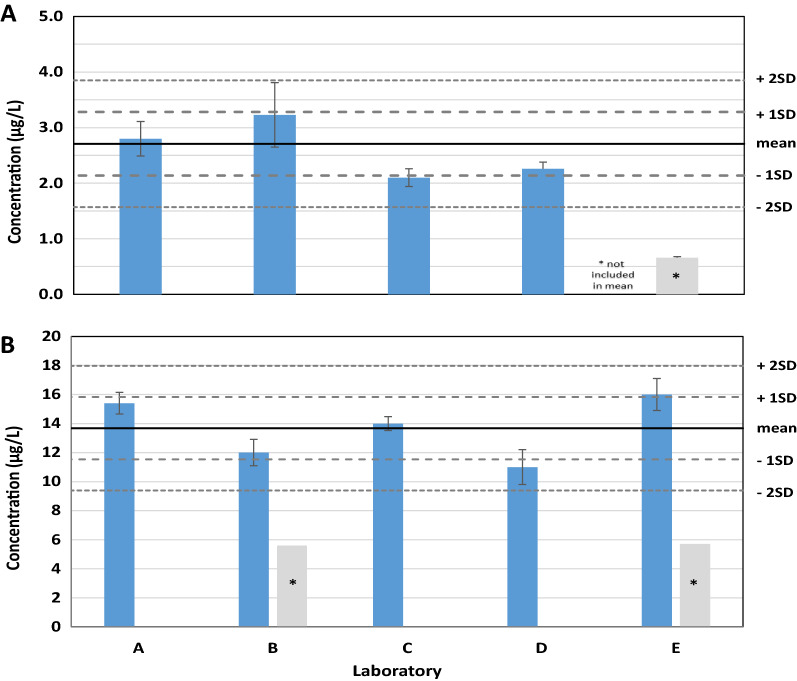
Water solubility results. **A** Dodecahydrotriphenylene by generator column. **B** *n*-hexylcyclohexane by slow-stir. Error bars represent one standard deviation from replicate analyses. (asterisk) Initial values not included in mean

The water solubility results for each of the two test compounds measured using the respective water solubility methods are presented in Fig. 1. Detailed tabulation of the water solubility measurements from each of the participating laboratories is listed in Additional file 1: Table S3.

### Dodecahydrotriphenylene by column elution method

The mean dodecahydrotriphenylene water solubility of 2.6 µg/L (RSD 20%) was calculated based on results from four of the five labs. The low value of 0.66 µg/L reported by Laboratory E was more than three standard deviations less than the mean of the other four labs and excluded as a statistical outlier. There were no clear methodological differences to explain the apparent difference compared to the other labs. Errors related to measuring the water solubility of very hydrophobic compound such as dodecahydrotriphenylene usually tend to overestimate the true water solubility due to the presence of microcrystals or similar solid particles resulting in observed enrichment above true solubility [21]. In instances where measured values are less than true water solubility, as is the case for the 0.66 µg/L dodecahydrotriphenylene results reported by Laboratory E, several factors can be considered. The first is loss attributable during sampling and storage. Dodecahydrotriphenylene is not particularly volatile so loss due to this is unlikely. However, the compound is very hydrophobic so there is a possibility of loss through sorption to the sample vessel especially if the collected sample fractions were stored prior to extraction or if only a sub-sample was taken and the entire sample container was not solvent rinsed. The second reason for not achieving true water solubility is not reaching equilibrium during the column elution process. The most obvious issue would be performing the test at too great of a flow rate through the column. This was not likely the case for Laboratory E as the flows they reported were actually slightly less than those reported by the other labs. However, there is always a risk of “channeling” whenever a packed column is used [22]. This results when a packed column develops micro-channels which allow the column eluent to bypass the bulk of the packing, in this case neat dodecahydrotriphenylene. This may result in inadequate contact time and yields lower measured water solubility than expected. Since Lab E used the direct packing method, there is the possibility that the crystalline structure of the lot of dodecahydrotriphenylene they used was too irregular for a uniform column packing. For the four labs that reported good agreement of measured dodecahydrotriphenylene water solubility, two used the direct packing method (Labs A and D) while the other two (Labs B and C) used the guideline described procedure of dissolving the test compound in solvent followed by application to an inert support material.

**Table 4:** Comparison of measured water solubility with model predictions (µg/L)

Compound	This study 20 °C	SpARC (v4.2) 20 °C	EPISuite (25 °C)		ACD/Labs (25 °C) (v11.02)
			WSKOW (v1.41)	Fragments (v1.01)	
Dodecahydrotriphenylene	2.6 (2.1–3.2) n = 4	0.37	4.9	26	55
*n*-Hexylcyclohexane	14 (11–16) n = 5	8.8	124	61	19

Values in parenthesis represent range of measured water solubility values

### n-Hexycyclohexane by slow-stir method

The mean n-hexylcyclohexane water solubility of 14 µg/L (RSD 16%) was calculated based on results provided by each of the five labs. Laboratories B and E initially reported values significantly lower than the overall mean. Additional investigation highlighted the importance of harmonizing mixing speed, vessel size and test substance loading between laboratories. *n*-Hexylcyclohexane is also very volatile and special care and attention needs to be observed when sampling, transferring and storing volatiles from aqueous systems to mitigate losses. When sampling volatile organics in water for analysis using vapor phase techniques (e.g. headspace, purge-and-trap, headspace SPME) water should be sampled with gas tight syringes with samples being be poured into the syringe barrel followed by sealing with the syringe plunger [23, 24]. The conventional syringe technique of drawing a sample with the plunger in place should be avoided as this imparts a slight vacuum causing loss of volatile organics. When sampling water for solvent extraction, the vial in which the sample is taken should be pre-filled with the necessary volume of organic solvent to capture the volatile organics before they are lost to volatilization. The initial set of low results reported by Laboratories B and E were also very variable and did not demonstrate that equilibrium had been reached which is a requirement of the slow-stir method. Both Laboratories B and E demonstrated equilibrium was reached with good reproducibility in their second attempts (Additional file 1: Figure S2). It is not possible to cite which of the harmonized parameters was responsible for the improved results but was likely a combination of factors including sample handling and confirming the slow-stir systems had reached equilibrium.

### Measured versus modeled water solubility values

Table 4 compares the mean measured water solubilities of dodecahydrotriphenylene and *n*-hexylcyclohexane determined in this study with predictions from commonly used quantitative structure–property relationship models. The different models have fundamentally different calculation methods and likely different calibration datasets. Prior comparisons to poorly water soluble constituents showed better agreement with SPARC, generally, but still varied by more than a factor of 5 in some cases [13]. It is likely that many of the existing QSAR models are under-represented in the calibration datasets, and therefore comparisons have some limited predictive capability. This highlights the need for high quality datasets based on reliable test methods.

## Discussion

The inter-laboratory comparison of the paired slow-stir method—*n*-hexylcyclohexane (liquid) versus generator column—dodecahydrotriphenylene (solid) demonstrates that the slow-stir water solubility method yields results with slightly better reproducibility than the existing column elution method. Both test compounds qualify as difficult-to-test due to their very low solubility. *n*_Hexylcyclohexane may be considered even more challenging due to its volatility. This study also reaffirms that difficult-to-test substances are just that, presenting a significant challenge even to laboratories experienced with well-established water solubility techniques, such as the column elution method. Despite having sufficiently sensitive analytical methods and prior experience with trace analysis in environmental media, a portion of the labs produced results, at least initially, which were significantly lower than the overall mean for the respective test compounds. The dodecahydrotriphenylene water solubility results from the single attempt from one of the five labs was ultimately excluded as a statistical outlier. This occurred despite the generator column technique being a well-established method dating back to several decades to at least 1978 [21] and thoroughly described in the regulatory test guideline dated 1995 [7].

One technical advancement from the present study relating to the existing column elution method is an alternative means of packing the generator column with neat solid or crystalline test substance. This modification avoids the onerous, solvent-use practice described in the current test guideline. After excluding the single results from one of the labs, results from packed column testing produced results that were similar to solvent-prepared generator columns. This adaptation results in measured concentrations that are comparable in magnitude and variability to the more conventional solvent-based technique. However, the excluded column elution result was from a lab packing the column directly with test compound. If the low measurement was attributable to channeling, it may have been the result of less than uniform particles of the test compound. Those applying the direct-packing technique are cautioned as to the potential channeling risks which may result in measuring lower than actual water solubility. Further evaluation is needed in streamlining the column packing in this regard including guidance on the uniformity of the test compound particles.

The slow-stir technique is a more recent water solubility method specifically applied to difficult-to-test liquid test substances. Direct experience with this technique is limited among testing labs and there is not a common established protocol being applied. The few published [6, 11–13] works describing the slow-stir method vary in their level of specific experimental details. Also, *n*-hexylcyclohexane is particularly volatile with a high Henry’s law constant making sampling and handling of samples particularly critical. The appropriate sample handling of very volatile difficult-to-test substances is of separate, but related, importance to this type of physical–chemical and environmental fate and effects testing. Extending key guidance for successful implementation of this method included recommendations on sample handling and agitation of the test solutions, which resulted in greater agreement between laboratories. Recommendations were made with regard to mixing speed (optimized at 80–250 rpm), vessel size (2 L minimum) and vessel headspace (preferably less than 10% of vessel volume). Also, best practices were shared for sample handling of volatile organics in water which included sampling with no headspace, minimizing or eliminating sample transfers and extracting or analyzing the samples the same day they are taken with no intermittent sample storage. During testing, it appeared that consistent application of the method with regard to vessel size and headspace, mixing speed and best practices for sampling volatile hydrocarbons in water improved agreement between laboratories. For example, the initial n-hexylcyclohexane results from Labs B and E were 5.6 µg/L and 5.7 µg/L, respectively. Their values increased two to three times to 12 µg/L and 16 µg/L, respectively, upon incorporating the suggested practices of mixing rate, loading and sample handling. Ultimately, these solubility methods are demonstrated to achieve reproducible results among participating laboratories and are considered fit-for-purpose.

The preliminary ring trial results reported here coupled with previous publications [6, 11–13] using the technique, demonstrate that the slow-stir water solubility method merits consideration for inclusion in the current water solubility test guidelines. The five laboratories applying the method to a single difficult-to-test compound, *n*-hexylcyclohxane, ultimately generated results with good inter-laboratory reproducibility. The variability across the five labs was actually slightly less than the variability recorded across the same labs applying the existing generator column method to a separate but equally challenging difficult-to-test solid compound, dodecahydrotriphenylene. The results demonstrated that acceptable reproducibility was attained with consistent test method application and careful sample handling. Earlier work described by Toll’s et al*.* [12] and Letinski et al*.* [11, 13] demonstrated good agreement in the application of the slow-stir technique between their respective labs across a common subset of six alkanes spanning water solubilties of < 1 µg/L to approximately 200 µg/L. Consistent with previous findings [13], the SPARC structure–property model was closest in predicting measured water solubility of liquid hydrocarbons, which includes *n*-hexylcyclohexane measured in this study. In this study, the EPIWIN WSKOW model was the best predictor of the solid dodecahydrotriphenylene water solubility. The authors could not identify any previously reported measured water solubility for dodecahydrotriphenylene.

A more comprehensive round robin test program with an expanded number of participants and evaluation of multiple, difficult-to-test liquid substances is recommended to support inclusion of the slow-stir method in the current water solubility test guidelines. Based on this study, previous publications and the authors’ prior experience with the slow-stir technique, a protocol should include the following:

1. All glass aspirator bottles or equivalent should be used with a sampling port or spigot located toward the bottom of the vessel (for test compounds with density < 1 g/mL).
2. The minimum vessel size should be 2 L and have an initial headspace of no more than 10% of the water volume. Vessel size is ultimately dictated by the volume of sample required for extraction and analysis.
3. In the case where smaller volume vessels (i.e. 2 L) are employed and the test compound is particularly volatile, individual slow-stir vessels should be established for each sampling interval in order to maintain small, consistent headspace volume.
4. The vessel should be tightly sealed with either Teflon™ screw-plugs, glass stoppers or other inert, non-absorptive materials.
5. Test substance should be added at loadings of three to four orders of magnitude greater than the expected water solubility. A minimum loading of 1 mg/L and maximum loading of 100 mg//L is recommended. Prior to removing samples at weekly intervals, the analyst should confirm that excess free liquid is visible on the water surface confirming that both the water and vapor phases are saturated.
6. Stirring rates between approximately 80 and 250 rpm, depending on the vessel size and water volume. The rate should be sufficient to impart a small dimple (< 0.5 cm) of the test substance below the water surface.
7. Water samples should be extracted and/or analyzed the day they are sampled from the slow-stir vessels. Because of the test compound hydrophobicity, and possibly volatility, water samples stored beyond the sampling day should be avoided as the substance may be lost through adsorption to the sample container or via volatility.
8. The highest quality reagent grade water (glass, distilled, double distilled, Milli Q) available should be used. Water should be poisoned with 50 mg/L of mercuric chloride or otherwise sterilized prior to initiation. This will prevent biodegradation of dissolved test compound in the water column which is especially problematic for substances that are readily biodegradable and have extremely low solubility where extended mixing times are needed to reach equilibrium.

A proposed addition of the slow-stir water solubility method to the existing OECD 105 Water Solubility test guideline is included in the Additional file 1. It is recommended that an expanded ring test include multiple difficult-to test compounds including a volatile and semi-volatile liquids plus a liquid compound with density greater than one gram per liter.

## Supplementary Information

**Additional file 1: Table S1. **Column elution test parameters among participating labs. **Table S2. **Slow-stir test parameters among participating labs. **Table S3.** Dodecahydrotriphenylene and n-hexylcyclohexane water solubility results.

## Data Availability

The data from the individual laboratories participating in the ring test described in this paper can be obtained by contacting the corresponding author.
