# Supplementary material for: Inter-laboratory comparison of water solubility methods applied to difficult-to-test substances
Source: BMC Chem. 2021 Sep 15;15(1):52. doi: 10.1186/s13065-021-00778-7 (PMC8442276; doi:10.1186/s13065-021-00778-7)
Supplement: Supplementary file 1 — Additional file 1: Table S1. Column elution test parameters among participating labs. Table S2. Slow-stir test parameters among participating labs. Table S3. Dodecahydrotriphenylene and n-hexylcyclohexane water solubility results. [file 13065_2021_778_MOESM1_ESM.docx]

**Additional Information**

**Table S1: Column elution test parameters among participating labs.**

| Laboratory | Test substance loading | Water | Flow Rate (mL/min) | | Volume  of each  fraction  (mL) |
| --- | --- | --- | --- | --- | --- |
|  |  |  | first  run | second  run |  |
| A | neat/no support | glass distilled | 0.8 | 0.4 | 5 |
| B | on support | demineralized water (Milli-Q System) | 0.6 | 0.3 | 10-20 |
| C | on support | double distilled | 0.5 | 0.25 | 25 |
| D | neat/no support | 18.2 MΩ, Milli-Q | 0.4 | 0.2 | 1.2 |
| E | neat/no support | double glass distilled | 0.33 | 0.17 | 5 |

**Table S2: Slow-stir test parameters among participating labs.**

| Laboratory | Total  vessel  volume  (L) | Water  volume  (L) | Headspace volume  (L) | Test  substance  loading  (mg/L) | Vessel  seal | Mixing  rate  (rpm) | Volume  removed  at each  sampling  interval | Sampling |
| --- | --- | --- | --- | --- | --- | --- | --- | --- |
| A | 4.3 | 4 | 0.3 | 10 | Terflon screw  plug | 80 | 100 | directly into 10 mL glass, gas-tight syringe then transfer to 20 mL HS vial |
| B | 0.97 | 0.8 | 0.17 | 38 | Glass plug sealed with parafilm | 250 | 20 |  |
| C | 2 | 2. | 0.3 | 10 | Polypropylene  screw plug | 100 | 50 | 10 mL into a beaker,  5 mL into a 10 mL glass vial +  internal standard solution,  extract with 1 mL hexane |
| D | 2 | 1.9 | 0.1 | 0.42 | plug | No  vortex | 15 | 20 mL glass vial, extraction  with 1.5 mL cyclohexane |
| E | 1 | 0.95 | 0.05 | 400 | HDPE plug | 200 | 30 | 5 mL directly into  headspace vials. |

**Table S3:**  **Dodecahydrotriphenylene and n-hexylcyclohexane water solubility results.**

| Laboratory | Dodecahydrotriphenylene | | | n-Hexylcyclohexane | | |
| --- | --- | --- | --- | --- | --- | --- |
|  | Mean Measured Concentration  (µg/L) | Standard Deviation  (µg/L) | Relative Standard  Deviation | Mean measured Concentration  (µg/L) | Standard Deviation  (µg/L) | Relative Standard  Deviation |
| A | 2.8^p^ | 0.31 | 11% | 15 | 0.74 | 4.9% |
| B | 3.2 | 0.58 | 18% | 12 (5.6^) | 1.4 | 12% |
| C | 2.1 | 0.16 | 7.4% | 14 | 1.2 | 8.6% |
| D | 2.3^p^ | 0.12 | 5.2% | 11 | 0.48 | 4.4% |
| E | 0.66^p,^* | 0.02 | 3.0% | 16 (5.7^) | 1.3 | 8.5% |
| overall | 2.6 | 0.52 | 20% | 14 | 2.2 | 16% |

p generator column packed directly with solid test compound

* Statistical outlier, not included in mean.

^ First attempt values not included in mean.

**SLOW-STIR METHOD**

**Principle**

1. The slow-stir method is intended for liquid test substances that have expected water solubilites of less than 1 mg/L and also have a density of less than 1 g/L at the test temperature (20^o^C). An aliquot of neat liquid test substance is placed on the surface of water contained in a glass vessel. The water is stirred very gently while the test substance remains on the water surface. As the stirring rate is minimal, the emulsification of the test substance and formation of microdroplets is avoided. Portions of the water are removed over time and the dissolved test substance concentration measured by a suitable analytical method. Once equilibrium has been reached, the measured dissolved test substance concentration represents the test substance’s true water solubility~~.~~

**Apparatus**

**2**.

- The apparatus consists of a glass aspirator bottle (Figure 1) equipped with sampling port at the bottom of the vessel. The vessel should have a minimal nominal volume of 2 L. The bottle should be able to be tightly sealed with an inert (e.g. Teflon, glass) stopper or cap. The contents of the bottle is maintained at constant temperature by using either a water jacketed vessel or by placing the bottle in a temperature controlled enclosure.
- To accommodate particularly volatile test substance where the increase in vessel headspace may be an issue over consecutive sampling intervals, replicate test systems should be established and designated for each sampling point. Replicate test systems may be similarly needed if the volume of water removed for extraction at each time interval is very large relative to the volume of the vessel.
- Double distilled, glass distilled or other ultra-high purity laboratory water. Optional - water can be sterilized with 50 mg/L of mercuric chloride or other means that do not alter the test substance, for those test substances with extremely low solubility that may biodegrade over lengthy equilibration periods.
- Glass syringes or pipettes for adding the test substance directly to the water surface.
- Stir plate and Teflon or glass coated stir bars.
- Suitable analytical instrumentation and sample extraction supplies for analysis of the dissolved test substance in water

**Figure 1**

**
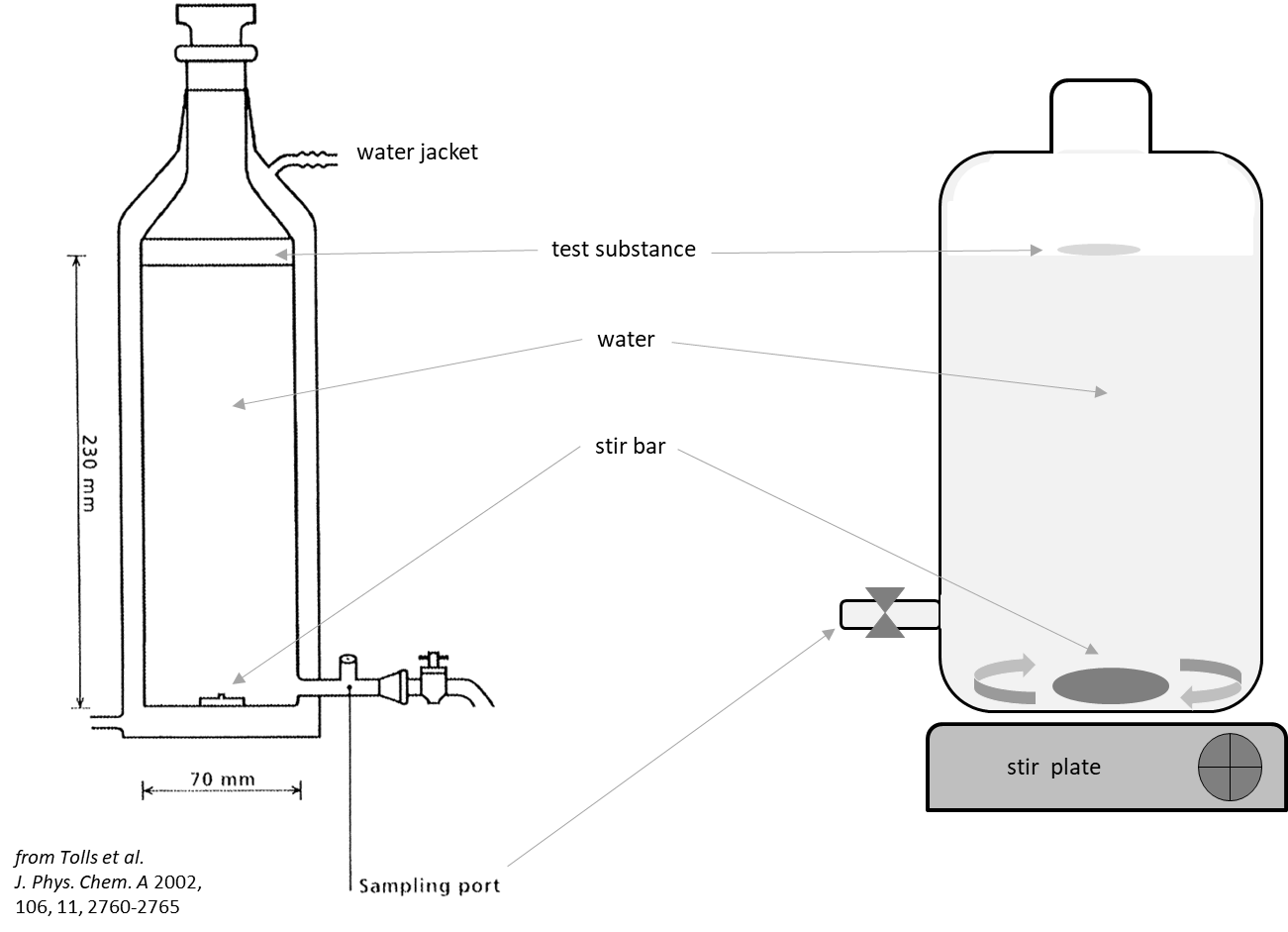
**

**Procedure**

1. The test vessel containing a stir bar and positioned on a stir plate should be filled with reagent grade laboratory water (glass or double distilled) to a height approximating the taper at the top of the bottle. The test substance should be added at a loading of approximately three orders of magnitude greater than the expected water solubility. Estimations of a test substance’s water solubility can be obtained using one of the commonly available structure-property models (e.g. SPARC, EPIWIN). The minimal loading should be 1 mg/L and the maximum loading should be 100 mg/L. Test substance loading is particularly critical for very volatile liquid test substances and should be sufficient to saturate both the water and vapor phases of the test system. The water in the test vessel should be permitted to come to test temperature prior to addition of the test substance. A parallel control system should be established with no test substance added. The test substance is added to the water surface by syringe or pipette, taking care to apply it directly to the water surface. The vessel is sealed and slow-stirring is initiated at a rate (80- 250 rpm, depending on vessel size) just sufficient to impart a slight vortex depth not exceeding two centimeters below the water surface.

**Sampling and Analysis**

**4.** The test system should be allowed to stir for a minimum of seven days prior to the first sampling and continue at approximately weekly intervals until the test substance concentration in water appears to have plateaued. At each sampling period, stirring is stopped for approximately one hour prior to sampling. The observation that free test substances is present on the water surface should be recorded. The first sample portion (ca. 50-100 mL) collected at each interval should be discarded so as to avoid sampling of the spigot’s dead volume. Sufficient water sample volume should be collected at each interval to provide for at least triplicate sample analysis plus an additional separate portion for pH determination. As the slow-stir method is designed to accommodate very hydrophobic test substances with very low water solubility, water samples should be extracted and/or analyzed as promptly as possible following sampling, preferably the day samples are taken. It is advised not to store water samples without solvent extraction. For volatile test substances, appropriate sample handling techniques must be applied during sampling and analysis. Stirring of the test systems is resumed upon the completion of weekly sampling. An analytical method should be applied that demonstrates adequate sensitivity and test substance specificity to quantify the dissolved test substance in water. Examples of such methods are: gas chromatography, liquid chromatography or spectrophotometry.

**Data**

**5.** Results should be obtained and reported for a minimum of three consecutive sampling intervals and include individual and mean measured concentrations along with relative standard deviations (RSD). Water solubility shall be reported as the mean concentrations from samples measured across a minimum of two connective sampling intervals, where the concentrations appear to have reached a saturation plateau and are no longer significantly increasing. For those samples used for calculation of the water solubility mean, the RSD across replicates at each sampling interval shall be no greater than 20%. The RSD across all of the samples used for the reported water solubility shall be no greater than 30%.

**Reporting**

**6.** The test report must include the following information:

- predicted water solubility obtained from a structure-property model and the model used for the estimation
- chemical identity and impurities (preliminary purification step, if any) and the substance’s density and physical state
- description of the slow stir test system including the vessel volume and reagent grade water used
- the loading (mg/L) of test substance in the test system
- the individual and mean measured concentrations of the test substance in water along with the relative standard deviations at each sampling interval
- the individual and mean reported water solubility concentrations with the relative standard deviation across all of the individual samples from which the reported water solubility concentration is calculated
  - the temperature of the test system during the study
  - the pH of the water sampled from the test and control system at each sampling interval
  - observation that free, excess test substance was visible on the test system surface at each sampling interval
  - complete description of the analytical method used including a description of the sample extraction method, if applicable
  - evidence of any chemical instability of the substance during the test
  - all information relevant for the interpretation of the results, in particular with regard to impurities and physical state of the substance

**Literature**

Determining the water solubility of difficult-to-test substances: A tutorial review. H. Birch, A.D. Redman, D.J. Letinski, D.Y. Lyon, P. Mayer, 2019Analytica Chimica Acta. 1086, 16-28. <https://doi.org/10.1016/j.aca.2019.07.034>

OECD 105, OECD Guideline for the Testing of Chemicals - Water Solubility,

1995, pp. 1e8, <https://doi.org/10.1787/9789264070684-en>

US EPA, Product properties test guidelines. OPPTS 830.7860 Water Solubility (Generator Column Method), 1996, pp. 1-17.

Octanol/water partition coefficients and water solubilities of phthalate esters, J.J. Ellington, J. Chem. Eng. Data 44 (1999) 1414e1418. <https://doi.org/10.1021/je990149u>

Slow-stir water solubility measurements of selected alcohols and diesters, D.J. Letinski, M.J. Connelly, D.R. Peterson, T.F. Parkerton, Chemosphere 48 (2002) 257e265, https://doi.org/10.1016/S0045-6535(02)00086-3

Aqueous Solubility_Molecular size relationships: a mechanistic case study using C10- to C19-alkanes, J. Tolls, J. Van Dijk, E.J.M.M. Verbruggen, J.L.M.M. Hermens, B. Loeprecht, G. Schüürmann, J. Phys. Chem. A 106 (2002) 2760e2765, <https://doi.org/10.1021/jp011755a>

Water solubility of selected C9-C18 alkanes using a slow-stir technique: comparison to structure - property models,D.J. Letinski, T.F. Parkerton, A.D. Redman, M.J. Connelly, B. Peterson, Chemosphere 150 (2016) 416e423, <https://doi.org/10.1016/j.chemosphere.2015.12.038>
